# Molecular Characterization of Rotifers and Their Potential Use in the Biological Control of *Biomphalaria*

**DOI:** 10.3389/fcimb.2021.744352

**Published:** 2021-09-21

**Authors:** Datao Lin, Suoyu Xiang, Benjamin Sanogo, Yousheng Liang, Xi Sun, Zhongdao Wu

**Affiliations:** ^1^Department of Parasitology, Zhongshan School of Medicine, Sun Yat-Sen University, Guangzhou, China; ^2^Provincial Engineering Technology Research Center for Diseases-Vectors Control, Key Laboratory of Tropical Disease Control, Ministry of Education, Guangzhou, China; ^3^Jiangsu Provincial Key Laboratory on Parasite and Vector Control Technology, Jiangsu Institute of Parasitic Diseases, Wuxi, China

**Keywords:** *Biomphalaria straminea*, *Biomphalaria glabrata*, *Schistosoma mansoni*, rotifer, *Philodina*, biocontrol strategy

## Abstract

**Background:**

Schistosomiasis is one of the most important tropical parasitic diseases worldwide. *Biomphalaria straminea*, the intermediate host of *Schistosoma mansoni*, has invaded and spread to Southern China since 1974 and may pose enormous threats to public health. Controlling intermediate host snails is an effective strategy in schistosomiasis intervention. However, the only effective chemical molluscicide, niclosamide, currently recommended by WHO may cause environmental pollution, loss of biodiversity, and high costs. Thus, to counter intermediate hosts, a sustainable and environmentally friendly tool is urgently needed. Here, we conducted field investigations to collect and identify a potential snail competitor rotifer and evaluated its molluscicide effect.

**Results:**

In this study, we collected two samples of rotifers from Shenzhen. We found both red and black phenotypic *B. straminea* snails at the sampling sites. We identified the rotifer population as a species of the genus *Philodina* according to the amplification and phylogenetic analysis results of *coxI* gene. We found that rotifer exposure did not significantly affect the hatching rate of *B. straminea* eggs but promoted the killing of juvenile snails. Meanwhile, rotifer exposure did not significantly alter the fecundity of *B. straminea* quantified by the number of eggs per egg mass, the number of egg masses per snail, and the number of eggs per snail; but the snails exposed to rotifers showed lower fecundity performance than the control snails. Importantly, rotifer exposure could significantly affect the development of juvenile *B. straminea*, showing a smaller shell diameter of the exposed snails than that of the control snails. In addition, rotifer exposure affected the life span of *B. straminea* snails, showing a 16.61% decline in the average life span. After rotifer exposure, the *S. mansoni*-infected *B. straminea* snails died significantly faster than those without rotifer exposure. Similar findings were observed in *S. mansoni*-infected *Biomphalaria glabrata* snails. These results implied that rotifer exposure significantly promoted the mortality of *S. mansoni*-infected *B. straminea* and *B. glabrata*.

**Conclusions:**

Our study demonstrated the potential molluscicide effect of rotifers on intermediate hosts under laboratory conditions. Our findings may provide new insights into the development of biocontrol strategies for snail-borne disease transmission.

## Introduction

Schistosomiasis is one of the most important human parasitic diseases (Chitsulo et al., 2000), causing almost 240 million people infected worldwide, which may cause huge economic and social burdens globally (Colley et al., 2014). Among all human infected schistosomes, *Schistosoma mansoni* is the most widespread species. *S. mansoni* is distributed predominantly in South America, Africa, the Caribbean, and the Middle East (Crompton, 1999; Chitsulo et al., 2000; Colley et al., 2014). *Biomphalaria* snails, including *Biomphalaria glabrata* and *Biomphalaria straminea*, are the main intermediate hosts of *S. mansoni* (Colley et al., 2014). As an important intermediate host of *S. mansoni* (Coelho and Caldeira, 2016), the freshwater snail *B. straminea* has invaded Hong Kong, China, since 1974 and has spread widely in South China (Meier-Brook, 1974; Lin et al., 2020). In addition, this invasive snail can also transmit the zoonotic parasite *Angiostrongylus cantonensis* (Xu et al., 2019; Zhu et al., 2019). Considering the potential risk of transmission of *S. mansoni* and threats to human health in China (Colley et al., 2014; Lin et al., 2020), it is necessary to pay more attention to monitoring and controlling *B. straminea* with close surveillance and control strategies.

The strategy of controlling intermediate hosts has been proven to be an effective approach to interrupt the transmission of *S. mansoni* (Lardans and Dissous, 1998). The application of chemical molluscicides is a major strategy for snail control. Niclosamide is the only molluscicide recommended by the WHO (Yang et al., 2010). However, the environmental effects, high toxicity to non-target organisms, and high costs in most endemic countries have hampered the widespread use of chemical molluscicides (Ekabo et al., 1996; Oliveira-Filho and Paumgartten, 2000). In addition, the application of niclosamide may induce resistance (Dai et al., 2015). To achieve the UN Sustainable Development Goals (SDGs), alternative tools for intermediate hosts and schistosomiasis control are urgently needed. In recent years, biocontrol strategies have attracted significant research attention due to their low toxicity and environmentally friendly features (de Oliveira et al., 2004; Soberon et al., 2013; Wei et al., 2017). Therefore, low toxicity and environmentally friendly tools are urgently needed and suited for invasive snail control.

There is a vast amount of zooplankton in rivers and oceans. As an important type of zooplankton, rotifers are widely distributed in freshwater bodies (Gilbert, 2017). Rotifers are an important food source of fishes in aquaculture (Stelzer, 2009; Dabrowski and Miller, 2018) and can also be indicators of environmental toxicity (Stelzer, 2009; Dabrowski and Miller, 2018; Colvin et al., 2021; Xu et al., 2021) and water quality (Jose et al., 2008; Picapedra et al., 2021). Nevertheless, rotifers can promote mortality by affecting the ingestion of shrimp (Yan et al., 2004; Yan et al., 2007) and cause tissue injury and fish death by attaching to the gills (Imai et al., 1991; Xu et al., 1999; Xu et al., 2000). Mass rotifers may rob food and nutrition from aquatic animals, inducing unhealthy status and mortality among aquatics (Meyabeme et al., 2010; Reyes-Prieto et al.2014; Ranasinghe and Amarasinghe 2020). *B. straminea* and *B. glabrata* are important freshwater snails and invasive species globally. In addition, the identification of microbiota as food competitors, such as rotifers, could be a potential additional tool for mosquito control (Ranasinghe and Amarasinghe, 2020). However, whether rotifers can be competitors of freshwater snails is unclear. Few studies have focused on controlling intermediate hosts by rotifers. Therefore, we hypothesized that rotifers could affect the development and survival of *Biomphalaria* snails.

In the present study, we collected rotifer samples from field studies in South China from 2016 to 2017 and investigated the influence and survival of *Biomphalaria* snails affected by rotifer exposure. Our findings may promote the development of biocontrol strategies for intermediate hosts.

## Methods

### Sample Collection

To collect the rotifer samples in Guangdong Province, we conducted systematic field surveys from 2016 to 2017. We collected about 100 snails from each sampling site. We found some rotifers were attaching to the surface of the *B. straminea* shell. Then, we collected samples and transferred alive rotifers to the laboratory. The name of the locality, Global Positioning System (GPS) coordinates, and date were recorded. We took pictures of the surroundings using a camera. The living specimens were maintained under laboratory conditions. We finally preserved several samples in 95% ethanol and stored them in −80°C for further processing.

### DNA Extraction

We removed the shell from the snail before genomic DNA extraction. Total DNA was extracted separately from approximately 30 mg of head–foot or the entire rotifer. All samples were individually crushed using a bead mill in an enzyme-free Eppendorf tube with 1-mm-diameter inox beads (Qiagen, Germany). After removing the beads, we extracted total DNA using the hipure DNA mini kit (Magen, China) as previously described (Lin et al., 2020). Briefly, genomic DNA was extracted according to the protocol of the kit, and finally, total DNA was suspended in 30 μl of nuclease-free buffer and stored at −80°C until further processing. The DNA quality and quantity were examined using a NanoDrop instrument (Thermo Fisher Scientific, USA).

### Amplification and Sequencing

The DNA samples were amplified for identification as described in the previous study (Lin et al., 2020). The universal cytochrome oxidase subunit (*cox*) I primer set for rotifer identification was used: LCO1490 5′-GGTCAACAAATCATAAAGATATTGG-3′ and HCO2198 5′-TAAACTTCAGGGTGACCAAAAAATCA-3′. The PCR amplification system for the target gene comprises 1 μl of cDNA, 12.5 μl of a mixture, 1 μl of forward primer, 1 μl of reverse primer, and 9.5 μl of double deionized water. The PCR cycling conditions were carried out: initial denaturation step at 94°C for 5 min followed by 30 cycles of 94°C for 45 s, 48°C for 45 s, and 72°C for 45 s with a final extension step at 72°C for 10 min. In addition, The universal *coxI* primer set was also used for the *Biomphalaria* species identification. The PCR conditions for the marker amplification were performed: denaturation at 94°C for 5 min, 30 cycles of 94°C for 50 s, 55°C for 50 s, 72°C for 50 s, and final extension at 72°C for 10 min. The PCR products were detected on 3% agarose gel electrophoresis and purified according to the protocol of the Qiagen gel extraction kit (Qiagen, Germany). The purified PCR products were sequenced on an ABI-3730 platform (Applied Biosystems) by the Majorbio company (Guangzhou, China).

### Phylogenetic Analysis

The sequences obtained from sequencing and the National Center for Biotechnology Information (NCBI) databases (https://www.ncbi.nlm.nih.gov/) were aligned and concatenated by the neighbor-joining method using the molecular evolutionary genetics analysis (MEGA) 7 (Kumar et al., 2016). We performed the parsimony analysis by generating 1,500 bootstrap replicates.

### Maintenance of the Snails in the Laboratory

The *Biomphalaria* snails were raised under laboratory conditions as described in the previous study (Lin et al., 2020). Each snail was exposed to 10 *S. mansoni* miracidia. The procedures for infecting snails with miracidia were described in the previous study (Keiser et al., 2014). The *S. mansoni*-exposed snails were maintained with shading treatment. The infection rate was measured as described in the previous study (Fernandez and Thiengo, 2002). The release of cercaria from *Biomphalaria* was previously described (Lin et al., 2020).

### Exposure Experiment Design

To investigate the effect of rotifers, we used eggs, juvenile, and mature snails to perform further experiments. i) We randomly divided eggs or snails on the same developmental stage into two groups: normal snails being exposed with or without rotifers. ii) *S. mansoni* miracidia-infected snails were also randomly divided into two groups: snails being exposed with or without rotifers. We selected the 2-week-old *Biomphalaria* snails for *S. mansoni*-infected experiments. After *S. mansoni* miracidia exposure experiments, both the exposed and unexposed snails were maintained under the same conditions. The survival rate was measured. The snail releasing *S. mansoni* cercaria was considered an infected snail.

### Influence of Parameters Measured

The fecundity (the number of eggs per egg mass, the number of egg masses per snail, and the number of eggs per snail) and fertility (rate of eggs hatched per mass) were measured as previously described (Costa et al., 2004). The number of hatching embryos was examined in 2 weeks, and subsequently, the egg hatchability was calculated. The survival and growth rates (shell diameter) were measured. The snails being measured for the shell diameter were randomly picked out from the alive juvenile snails.

### Statistical Analysis

We calculated the results using GraphPad Prism version 6.0 (GraphPad Software, USA). Data are expressed as the mean ± standard error of the mean (SEM). The differences between groups were analyzed by Student’s *t*-test using SPSS 19.0 software (SPSS Inc., USA). The survival rates between groups were analyzed using the chi-square test. ^*^ *p* < 0.05 was considered statistically significant.

## Results

### Sampling Site Study

**Figure 1:**
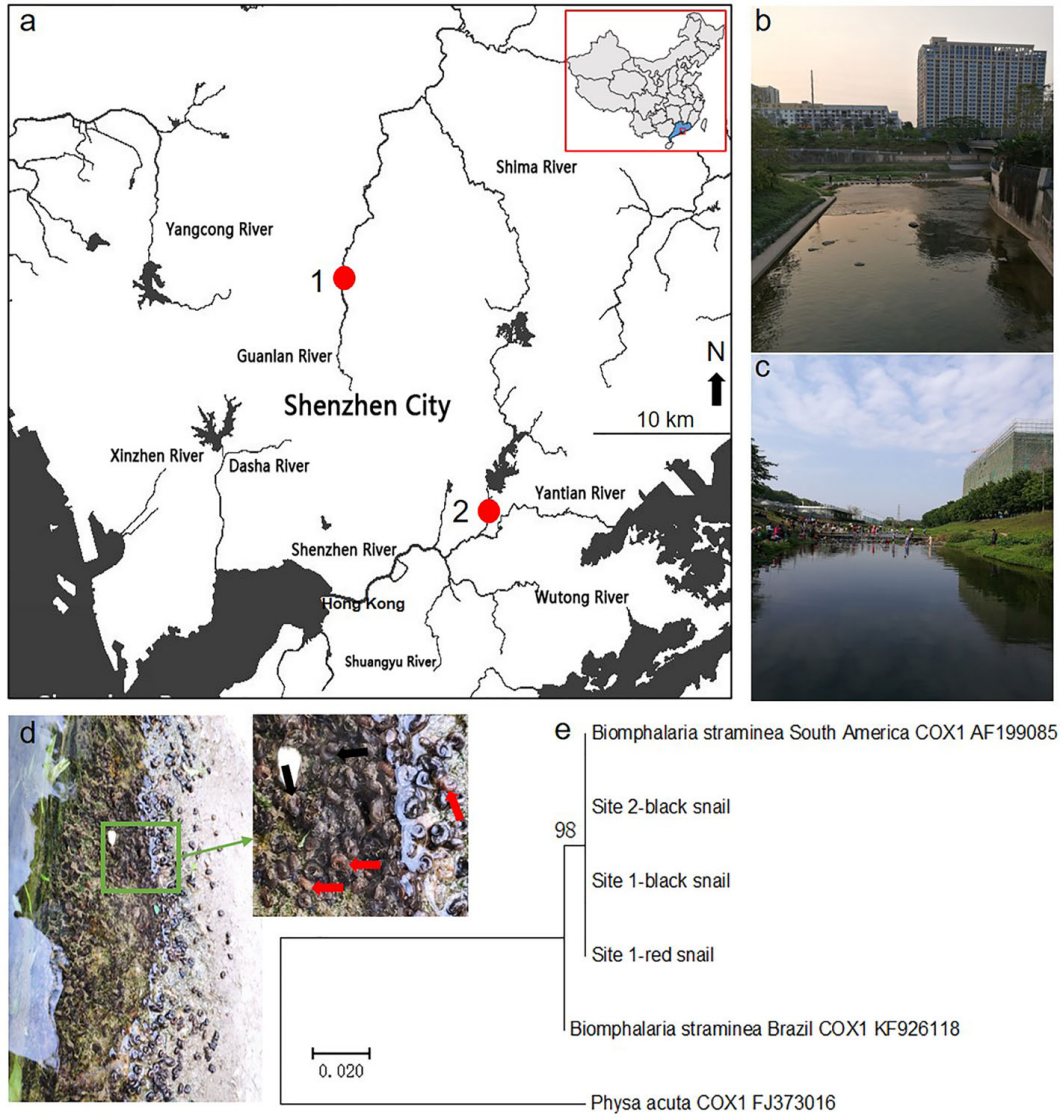
Sampling site study. The map showing sampling sites in Shenzhen **(A)** and pictures of rotifer habitats in the Guanlan River **(B)** and Donghu Park **(C)**. The red spot represents the rotifer sampling site. Both red and black phenotypic *Biomphalaria* snails were found in sampling site 1 **(D)**, and black snails were collected in site 2. The red arrow shows the red *Biomphalaria* snail observed, and the black arrow shows the black *Biomphalaria* snail observed. **(E)** Neighbor-joining tree constructed based on the K2P+G model for *coxI* sequences obtained from National Center for Biotechnology Information (NCBI) database and *Biomphalaria* samples collected from Shenzhen. This map was created using ArcGIS.

We found that some rotifers were attached to the surface of the shell of *Biomphalaria* snails in field studies. Then, we collected rotifer samples from the sites of the Guanlan River (22°40′18" N and 114°2′25" E) and Donghu Park (22°33′26" N and 114°8′38" E) in Shenzhen in South China (**Figure 1A**). Pictures of the surroundings of these sampling sites are shown (**Figures 1B, C**). In addition, maximum-likelihood trees showed that both the red and black phenotypic *Biomphalaria* snails collected from Shenzhen were similar to the South American *B. straminea* strain (**Figures 1D, E**).

### Species Identification of Rotifer

**Figure 2:**
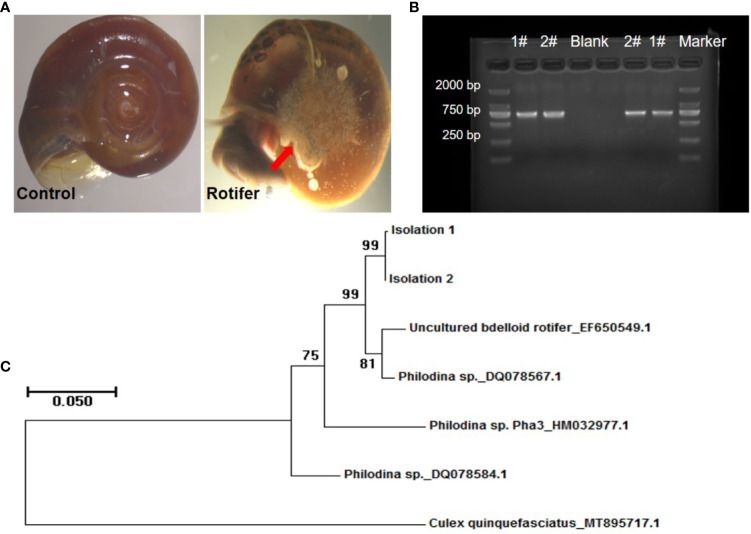
Amplification and phylogenetic analysis of rotifers collected in fields. **(A)** Pictures of rotifers attaching to navels of *Biomphalaria straminea* snail compared with control snail. The red arrow shows the rotifers observed. **(B)** Picture of PCR amplification based on *coxI* sequence extracted from rotifers (Lane 1 and 2). #, Rotifer isolation. Negative control (Blank). Marker: about 750 bp. **(C)** Neighbor-joining tree constructed based on K2P+G model for *coxI* sequences obtained from National Center for Biotechnology Information (NCBI) database and rotifer samples (Isolations 1 and 2) collected from Shenzhen.

We found that the rotifers were mainly attached to the navels of *Biomphalaria* snails (**Figure 2A**). To determine the species identification of rotifers from sampling sites, *coxI* gene was amplified and sequenced for phylogenetic reconstruction. The PCR fragments of *coxI* gene of rotifer were amplified and resolved in an agarose gel (**Figure 2B**). The five referenced sequences of mitochondrial genes obtained from the NCBI database included EF650549.1 (uncultured *bdelloid rotifer*), DQ078567.1 (*Philodina* sp.), HM032977.1 (*Philodina* sp. Pha3), DQ078584.1 (*Philodina* sp.), and MT895717.1 (*Culex quinquefasciatus*) (**Figure 2C**). We found that our samples (Isolations 1 and 2) clustered on the same branch, similar to the branches of uncultured *bdelloid* rotifers and *Philodina* sp. (**Figure 2C**). The sequence similarity between these two clusters was greater than 97% according to the BLAST results (https://blast.ncbi.nlm.nih.gov/Blast.cgi). These results showed that our rotifer samples belonged to the genus *Philodina*. Therefore, we named our rotifer samples collected from Shenzhen *Philodina* sp. sz1 and *Philodina* sp. sz2.

### Rotifer Exposure Did Not Significantly Influence the Hatching Rate of *B. straminea* Egg Masses

**Figure 3:**
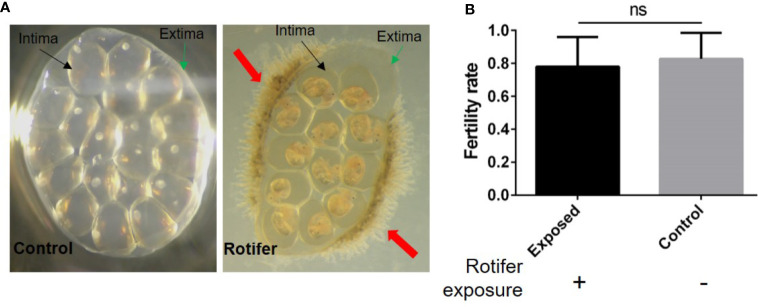
The effect of rotifer exposure on *Biomphalaria straminea* egg masses. **(A)** Pictures of rotifers attaching to the egg mass of *B straminea* compared with control snail (left). The green arrow shows the extima of the egg mass. The black arrow shows the intima of egg mass. The red arrow shows rotifers. **(B)** The difference of hatching rate of egg mass between with and without rotifer exposure. ns, Not statistically significant.

To investigate the effect of rotifers on the hatching rate of *B. straminea*, we randomly divided egg masses of *B. straminea* into two groups. We observed gelatinous intima and extima on the egg masses (**Figure 3A**). Rotifers were only attached on the edge of the *Biomphalaria* egg mass and segregated into eggs by the extima and intima (**Figure 3A**). We found no significant difference in the hatching rates between groups infected with (0.7839 ± 0.03658) or without (0.8267 ± 0.02927) rotifers, but on average, the hatching rate declined in the rotifer-infected masses (**Figure 3B**).

### Rotifer Exposure Affected the Development of Juvenile *B. straminea* Snails

**Figure 4:**
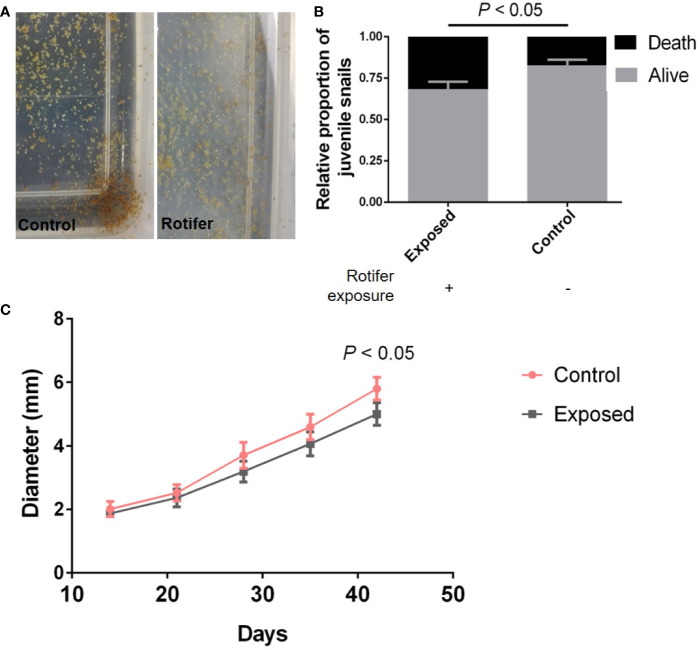
The effect of rotifer exposure on juvenile *Biomphalaria straminea* snails. **(A)** Pictures of juvenile *B. straminea* being exposed to rotifers (right) and control group with rotifer exposure (left). **(B)** The difference of survival rate of juvenile *B. straminea* between with and without rotifer exposure in 6 weeks. **(C)** The influence on the development of juvenile *B. straminea* exposed to rotifers. This result was quantified by the shell diameter of *B. straminea*.

As our results showed before, there was no significantly different effect on the hatchability. However, the required times for juvenile snails to hatch from the egg mass were not similar, ranging from 5 to 14 days. The juvenile snails hatching from egg masses were exposed to rotifers (**Figure 4A**), and the juvenile snails immediately become infected with rotifers after hatching. We found that the survival rate of juveniles in the infected group declined significantly compared with that of the control snails (**Figure 4B**). In addition, rotifers affected the development of juveniles, which showed a significantly smaller shell diameter than the control snails (**Figure 4C**).

### Rotifer Exposure Affects the Fecundity and Sexual Maturation Time of *B. straminea*

**Figure 5:**
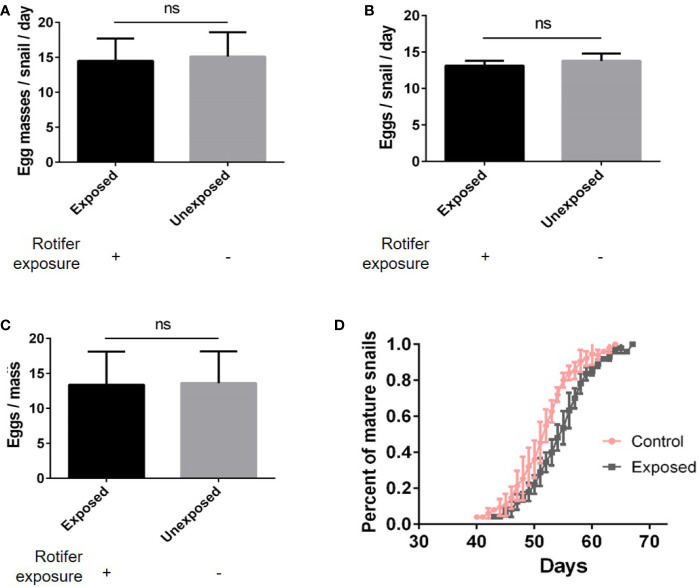
The effect of rotifer exposure on the fecundity of *Biomphalaria straminea*. **(A)** The difference in the number of egg mass per snail per day. **(B)** The difference in the number of eggs per snail. **(C)** The difference in the number of eggs per mass. **(D)** The difference in oviposition time of juvenile *B. straminea* snails exposed to rotifers or without rotifers. ns, Not statistically significant.

We found no significant differences in the number of egg masses per snail per day (**Figure 5A**), the number of eggs per snail (**Figure 5B**), or the number of eggs per mass (**Figure 5C**). However, rotifer exposure significantly affected the oviposition time of juvenile *B. straminea*, indicating that rotifers may delay the sexual development of *B. straminea* (**Figure 5D** and **Table 1**). Our study showed that there was no significant difference in the fecundity of snails infected with or without rotifers.

**Table 1:** The average differences in the oviposition time of *Biomphalaria straminea* exposed to rotifers or without rotifers (control group).

Item	Control (n = 64) Mean ± SEM	Exposed (n = 67) Mean ± SEM	Significance
Sexual maturation time (days)	51.97 ± 0.5953	54.60 ± 0.6341	*p* < 0.05

### Rotifer Exposure Affected the Life Span of *B. straminea* Snails

**Figure 6:**
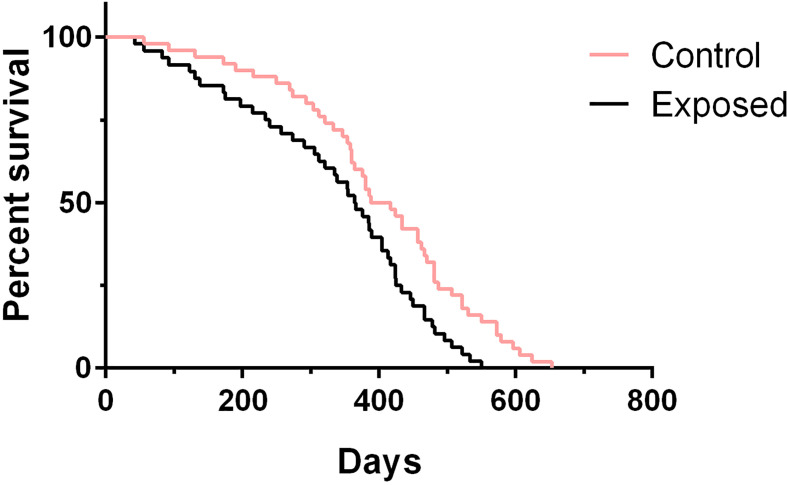
The survival analysis of *Biomphalaria straminea* exposed to rotifers or without rotifers (control group).

To test whether rotifer exposure could impact aging and affect life span, we treated juvenile *B. straminea* with rotifers. We measured the life spans of both exposed snails and control snails (without rotifer exposure). Since our previous results showed that rotifer exposure significantly affected the survival rate of juvenile *B. straminea*, we selected 5-week-old snails for further studies. We found that exposed *B. straminea* experienced dramatic life span shortening as compared with the control group (**Figure 6**, **Table 2**), showing a 16.61% decline in median life span after rotifer exposure.

**Table 2:** The average difference in the life spans of *Biomphalaria straminea* exposed to rotifers or without rotifers (control group).

Item	Control (n = 50) Mean ± SEM	Exposed (n = 48) Mean ± SEM	Significance
Life span (days)	401.0 ± 19.59	334.4 ± 19.70	*p* < 0.05

### Rotifer Exposure Affected the Survival of *S. mansoni*-Infected *B. straminea* and *B. glabrata* Snails

**Figure 7:**
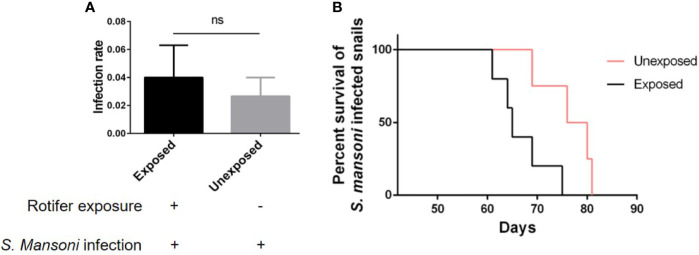
The effect of rotifer exposure on the survival of *Schistosoma mansoni*-infected *Biomphalaria straminea* snails. **(A)** Rotifer exposure did not significantly affect the infection rate of *B straminea* to *S. mansoni*. **(B)** Rotifer exposure decreased the survival rate of *S. mansoni*-infected *B. straminea*. Unexposed: the *S. mansoni*-infected snail was not exposed to rotifers. Exposed: the *S. mansoni*-infected snail was exposed to rotifers. ns, Not statistically significant.

As an intermediate host of *S. mansoni*, *B. straminea* plays an important role in the transmission of *S. mansoni*. Therefore, reducing the transmission risk of *S. mansoni* can be helpful for disease control. However, we found that rotifer exposure did not significantly alter the infection rate of *S. mansoni*-exposed *B. straminea* snails (**Figure 7A**). To conduct further experiments, we used positive snails that can release the cercaria of *S. mansoni*. After rotifer exposure, the *S. mansoni*-infected *B. straminea* died faster than the unexposed snails (**Figure 7B** and **Table 3**), showing a decline in the release time of cercaria from intermediate hosts.

**Table 3:** The average difference in the life span of *Schistosoma mansoni*-infected *Biomphalaria straminea* after rotifer exposure.

Item	Unexposed (n = 5) (Mean ± SEM)	Exposed (n = 4) (Mean ± SEM)	Significance
Life span (days)	66.80 ± 2.417	76.50 ± 2.723	*p* < 0.05

**Figure 8:**
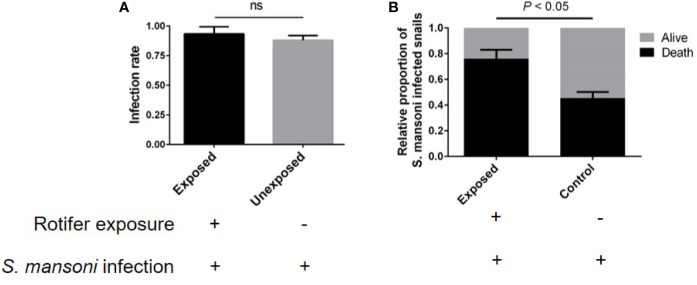
The effect of rotifer exposure on the survival of *Schistosoma mansoni*-infected *Biomphalaria glabrata* snails. **(A)** Rotifer exposure did not significantly affect the infection rate of *B. glabrata* to *S. mansoni*. **(B)** Rotifer exposure decreased the survival rate of *S. mansoni*-infected *B glabrata* in 15 weeks. ns, Not statistically significant.

*B. glabrata* is an important model organism for researching the interaction mechanism between *S. mansoni* and mollusks. We also detected the effect of rotifer exposure on the survival of *S. mansoni*-infected *B. glabrata*. We found that rotifer exposure did not significantly alter the infection rate of *S. mansoni*-exposed *B. glabrata* snails (**Figure 8A**). Since the infection rate was not 100% after *S. mansoni* miracidia exposure, we used positive snails that could release the cercaria of *S. mansoni* for further studies. We found that the *S. mansoni*-infected *B. glabrata* died significantly more than unexposed snails after 15 weeks of *S. mansoni* infection (**Figure 8B**). Rotifer exposure may accelerate the death of *Biomphalaria* snails infected with *S. mansoni*. Our results showed that rotifer exposure did not significantly alter the infection rate but significantly promoted the mortality of *S. mansoni*-exposed *Biomphalaria* snails, indicating the potential use of rotifer exposure on snail-borne disease transmission.

## Discussion

The freshwater snail *B. straminea*, which plays an important role in the transmission of *S. mansoni*, is one of the most widely distributed species in the genus *Biomphalaria* and originated from the southeastern part of South America (Colley et al., 2014; Yang et al., 2018). During the last decades, *B. straminea* has been reported in tropical countries, including Brazil, Paraguay, Argentina, Uruguay, Colombia, and Costa Rica (Lin et al., 2020). *B. straminea* was first reported to be introduced into Hong Kong in 1974 and has now spread to Shenzhen, Dongguan, Huizhou, and Puning in South China (Colley et al., 2014; Yang et al., 2018). Historically, China has been a non-endemic area for blood flukes of *S. mansoni*. However, with the increasing imported schistosomiasis cases in China (Dai et al., 2020; Wang et al., 2020) and the spread of the intermediate host (Meier-Brook, 1974; Lin et al., 2020), the potential risk of transmission of *S. mansoni* is increasing. Considering the potential threats to human health, we should pay more attention and make efforts to manage these snails. Controlling intermediate hosts is considered an effective approach to interrupt the transmission of *S. mansoni* and control snail-borne disease schistosomiasis (Lu et al., 2018). Using chemical molluscicides to control snails was the major strategy. However, chemical molluscicides such as niclosamide are highly toxic to other aquatic animals. Therefore, environmentally friendly tools are urgently needed for intermediate host control. In the present study, we detected potential biocontrol strategies for the intermediate host of *S. mansoni*.

We reported the isolation of populations of rotifers collected from Shenzhen for biocontrol tools to the intermediate host of *S. mansoni*. According to *coxI* gene sequence analysis, we positioned these rotifers within the genus *Philodina*. Since these identity levels are greater than the genomic definition of a species based on *coxI* gene, we proposed to name our rotifer samples collected from Shenzhen rotifer *Philodina* sp. sz1 and rotifer *Philodina* sp. sz2. As important zooplankton, rotifers are distributed in all kinds of water bodies, though mainly in freshwater bodies (Lu et al., 2018). Rotifers naturally coexist with aquatic organisms and are an important food source of fish and shrimp (Stelzer, 2009; Dabrowski and Miller, 2018). However, previous studies have shown that rotifers can also affect the development and survival of fish and shrimp (Imai et al., 1991; Xu et al., 1999; Xu et al., 2000; Yan et al., 2004; Yan et al., 2007). Yet whether rotifers affect the development and survival of Gastropoda, such as *B. straminea* snails, is unknown.

*B. straminea* has already spread to Hong Kong and Guangdong provinces in South China (Lin et al., 2020). Our findings revealed that both the red and black phenotypic *Biomphalaria* snails collected from Shenzhen were similar to the South American *B. straminea* strain, implying that these two sites exhibited two kinds of invasive freshwater snail phenotypes, *B. straminea*. The population level of the intermediate host *B. straminea* may be associated with the number of snails exposed to *S. mansoni* in the field: the more snails that exist, the more snails that are infected (de Souza et al., 1981; Fernandez and Pieri, 2001; Gandasegui et al., 2018). We found that rotifer exposure did not significantly affect the hatching rate of *Biomphalaria* eggs, and we hypothesized that a gelatinous membrane may cover the eggs, protecting them from pathogens. The hatching rate of *B. straminea* in our study was similar to that in previous studies (Scherrer et al., 1976; Costa et al., 2004) but was lower than that of *Biomphalaria pfeifferi* (Kengne-Fokam et al., 2016). In addition, rotifer exposure did not significantly affect the fecundity of *B. straminea*, showing no difference, but declines in the number of eggs per egg mass, the number of egg masses per snail, and the number of eggs per snail. We hypothesized that the reason for these declines was that there was enough food to supply snails and protect the fecundity of snails. The fecundity performance of *B. straminea* in our work was lower than that of *B. glabrata* (Rozemberg et al., 1992; Costa et al., 2004). These results suggested that the fertility of the genus *Biomphalaria* snails may be associated with genotype.

Our study revealed that rotifer exposure can significantly affect the development of *B. straminea* snails according to the shell diameter results, implying a potential influence on the reproduction and maturity of *Biomphalaria* snails. Previous studies revealed that there was an increase in growth inhibition in *S. mansoni*-infected snails (Looker and Etges, 1979; Meier and Meier-Brook, 1981; Cardoso and Coelho, 1990). Therefore, the growth of rotifer-exposed snails was similar to that of *S. mansoni*-infected snails. However, the association between growth alterations and parasite infection is unclear. Further studies on the mechanism of growth inhibition by rotifers are needed.

Rotifer exposure affected the survival rate of *B. straminea* snails, mainly juvenile snails. Importantly, rotifer exposure caused a significant decline in the average life span of *B. straminea* snails. Although previous studies have attempted to explore control strategies for intermediate hosts in China, they have mainly focused on chemical molluscicides, including salicylanilidate (He et al., 2017) and pyridylphenylurea derivatives (Wang et al., 2018). We focused on environmentally friendly tools to control intermediate hosts. Our findings suggested that rotifers may become a potential biocontrol tool for the intermediate host of *S. mansoni*. As one of the biocontrol strategies, pathogenic bacteria, including *Candidatus Paenibacillus glabratella* (Duval et al., 2015), *Bacillus thuringiensis* (Soberon et al., 2013), and *Beauveria bassiana* (Wei et al., 2017), have been further studied and have become potential alternative tools in disease intervention. Although the application of chemical molluscicides such as niclosamide is the most widely used method for snail control (Lardans and Dissous, 1998), we believe that environmentally friendly tools for intermediate hosts will be obtained with increasing research on biocontrol strategies.

Our work revealed that rotifers did not significantly affect the survival rate of *adult Biomphalaria* snails or the infection rates of *S. mansoni*-exposed snails. The susceptibility of the *Biomphalaria* snails mainly depends on their immune system, not foreign organisms (Hanington et al., 2010; Pila et al., 2016). Our findings demonstrated that rotifers promoted the killing of *S. mansoni*-infected *Biomphalaria* snails, including *B. straminea* and *B. glabrata*, implying that rotifer exposure may decrease the releasing cercaria of *S. mansoni* over time and contribute to disease control. Trematode parasites and their molluscan hosts produce antioxidants and oxidants to maintain the cellular redox balance, which may explain their survival in the late stage of parasite infection (Bayne et al., 2001; Bayne, 2009; Mourao et al., 2009). Rotifers not only grab food from other species but also receive foreign DNA from the animal kingdom, fungi, plants, and bacteria (Gladyshev et al., 2008; Boschetti et al., 2012; Szydlowski et al., 2015). Therefore, rotifer exposure may increase the burden on the survival of *Biomphalaria* snails and ultimately induce an imbalance. These results implied that rotifer exposure may interrupt the immune balance between *S. mansoni* and host snails, leading to snail mortality. However, the mechanisms of these findings are unclear, and further studies are needed.

## Conclusion

In our study, we identified a species of the genus *Philodina* rotifer collected from Shenzhen, South China. Rotifer exposure can alter the fecundity and significantly affect the fertility and life span of *B. straminea*, promote the death of juvenile snails, and significantly promote the mortality of *S. mansoni*-infected *B. straminea* and *B. glabrata*. Overall, our study demonstrated that rotifers may contribute to snail control and disease intervention by affecting the development and population quantity of *Biomphalaria* snails, in addition to *S. mansoni*-infected snails. Our results implied that rotifers may be a potential use and supplement in controlling snail-borne schistosomiasis transmission.

## Data Availability Statement

The data presented in the study are deposited in the GenBank repository, accession number (OK156495-OK156499). Please contact the author for additional data requests.

## Author Contributions

ZW, XS, and DL conceived and designed the study. DL carried out the experiments, prepared the manuscript, and handled the statistical analysis and interpretation of the data. DL, SX, BS, and YL critically revised the draft version of the paper. All authors contributed to the article and approved the submitted version.

## Funding

This work was supported by the National Key R&D Program of China (Nos. 2020YFC1200100 and 2016YFC1200500), the Natural Science Foundation of Guangdong Province (Nos. 2019A1515012068 and 2021A1515010976), the 111 Project (No. B12003), the Pearl River Nova Program of Guangzhou (No.201710010030), the Fundamental Research Funds for the Central University (No. 17ykpy09), the National Natural Science Foundation of China (Nos. 81802036 and 81871682), and the Natural Science Foundation of Guangdong Province (No. 2020A1515010896).

## Conflict of Interest

The authors declare that the research was conducted in the absence of any commercial or financial relationships that could be construed as a potential conflict of interest.

## Publisher’s Note

All claims expressed in this article are solely those of the authors and do not necessarily represent those of their affiliated organizations, or those of the publisher, the editors and the reviewers. Any product that may be evaluated in this article, or claim that may be made by its manufacturer, is not guaranteed or endorsed by the publisher.

## Abbreviations

SDGs, Sustainable Development Goals; *COX*, cytochrome oxidase subunit; GPS, Global Positioning System; MEGA, molecular evolutionary genetics analysis.

## References

[B1] Bayne C. J. (2009). Successful Parasitism of Vector Snail Biomphalaria Glabrata by the Human Blood Fluke (Trematode) Schistosoma Mansoni: A 2009 Assessment. Mol. Biochem. Parasitol. 165 (1), 8–18. doi: 10.1016/j.molbiopara.2009.01.005 19393158 PMC2765215

[B2] Bayne C. J. Hahn U. K. Bender R. C. (2001). Mechanisms of Molluscan Host Resistance and of Parasite Strategies for Survival. Parasitol. 123 (Suppl), S159–S167. doi: 10.1017/s0031182001008137 11769280

[B3] Boschetti C. Carr A. Crisp A. Eyres I. Wang-Koh Y. Lubzens E.. (2012). Biochemical Diversification Through Foreign Gene Expression in Bdelloid Rotifers. PloS Genet. 8 (11), e1003035. doi: 10.1371/journal.pgen.1003035 23166508 PMC3499245

[B4] Cardoso G. S. Coelho P. M. (1990). [Schistosoma Mansoni: Quantitative Aspects of the Fertility and Survival of Worms of Irradiated Cercariae (3 Krad), in Mice]. Rev. Inst Med. Trop. Sao Paulo. 32 (1), 28–35. doi: 10.1590/S0036-46651990000100005 2259829

[B5] Chitsulo L. Engels D. Montresor A. Savioli L. (2000). The Global Status of Schistosomiasis and its Control. Acta Trop. 77 (1), 41–51. doi: 10.1016/s0001-706x(00)00122-4 10996119 PMC5633072

[B6] Coelho P. Caldeira R. L. (2016). Critical Analysis of Molluscicide Application in Schistosomiasis Control Programs in Brazil. Infect. Dis. Poverty. 5 (1), 57. doi: 10.1186/s40249-016-0153-6 27374126 PMC4931695

[B7] Colley D. G. Bustinduy A. L. Secor W. E. King C. H. (2014). Human Schistosomiasis. Lancet. 383 (9936), 2253–2264. doi: 10.1016/S0140-6736(13)61949-2 24698483 PMC4672382

[B8] Colvin K. A. Parkerton T. F. Redman A. D. Lewis C. Galloway T. S. (2021). Miniaturised Marine Tests as Indicators of Aromatic Hydrocarbon Toxicity: Potential Applicability to Oil Spill Assessment. Mar. Pollut. Bull. 165:112151. doi: 10.1016/j.marpolbul.2021.112151 33601277

[B9] Costa M. J. Grault C. E. Confalonieri U. E. (2004). Comparative Study of the Fecundity and Fertility of Biomphalaria Glabrata (Say 1818) and Biomphalaria Straminea (Dunker 1848) in a Laboratory Through Self-Fertilization and Cross-Fertilization. Rev. Inst Med. Trop. Sao Paulo. 46 (3), 157–163. doi: 10.1590/s0036-46652004000300007 15286820

[B10] Crompton D. W. (1999). How Much Human Helminthiasis is There in the World? J. Parasitol. 85 (3), 397–403. doi: 10.2307/3285768 10386428

[B11] Dabrowski K. Miller M. (2018). Contested Paradigm in Raising Zebrafish (Danio Rerio). Zebrafish. 15 (3), 295–309. doi: 10.1089/zeb.2017.1515 29485943 PMC6037192

[B12] Dai S. M. Guan Z. Zhang L. J. Lv S. Cao C. L. Li S. Z.. (2020). Imported Schistosomiasis, China 2010-2018. Emerg. Infect. Dis. 26 (1), 179–180. doi: 10.3201/eid2601.191250 31855529 PMC6924890

[B13] Dai J. R. Li Y. Z. Wang W. Xing Y. T. Qu G. L. Liang Y. S. (2015). Resistance to Niclosamide in Oncomelania Hupensis, the Intermediate Host of Schistosoma Japonicum: Should We be Worried? Parasitology 142 (2), 332–340. doi: 10.1017/S0031182014000870 25003984

[B14] de Oliveira E. J. Rabinovitch L. Monnerat R. G. Passos L. K. Zahner V. (2004). Molecular Characterization of Brevibacillus Laterosporus and its Potential Use in Biological Control. Appl. Environ. Microbiol. 70 (11), 6657–6664. doi: 10.1128/AEM.70.11.6657-6664.2004 15528531 PMC525142

[B15] de Souza C. P. Rodrigues M. S. de Azevedo M. L. Araujo N. (1981). [Susceptibility of Populations of Biomphalaria Straminea (Dunker 1848) From Minas Gerais, to Schistosoma Mansoni Infection]. Rev. Inst Med. Trop. Sao Paulo. 23 (5), 212–216. 7323606

[B16] Duval D. Galinier R. Mouahid G. Toulza E. Allienne J. F. Portela J.. (2015). A Novel Bacterial Pathogen of Biomphalaria Glabrata: A Potential Weapon for Schistosomiasis Control? PloS Negl. Trop. Dis. 9 (2), e3489. doi: 10.1371/journal.pntd.0003489 PMC4342248 25719489

[B17] Ekabo O. A. Farnsworth N. R. Henderson T. O. Mao G. Mukherjee R. (1996). Antifungal and Molluscicidal Saponins From Serjania Salzmanniana. J. Nat. Prod. 59 (4), 431–435. doi: 10.1021/np960208r 8699187

[B18] Fernandez M. A. Pieri O. S. (2001). Infection by Schistosoma Mansoni Sambon 1907 in the First Four Months of Life of Biomphalaria Straminea (Dunker 1848) in Brazil. Mem Inst Oswaldo Cruz. 96 (Suppl), 185–192. doi: 10.1590/s0074-02762001000900029 11586448

[B19] Fernandez M. A. Thiengo S. C. (2002). Susceptibility of Biomphalaria Straminea (Dunker 1848) From Serra Da Mesa Dam, Goias, Brazil to Infection With Three Strains of Schistosoma Mansoni Sambon 1907. Mem Inst Oswaldo Cruz. 97 (Suppl 1), 59–60. doi: 10.1590/s0074-02762002000900013 12426596

[B20] Gandasegui J. Fernandez-Soto P. Muro A. Simoes B. C. Lopes D. M. F. Loyo R.. (2018). A Field Survey Using LAMP Assay for Detection of Schistosoma Mansoni in a Low-Transmission Area of Schistosomiasis in Umbuzeiro, Brazil: Assessment in Human and Snail Samples. PloS Negl. Trop. Dis. 12 (3), e6314. doi: 10.1371/journal.pntd.0006314 PMC5849311 29534072

[B21] Gilbert J. J. (2017). Non-Genetic Polymorphisms in Rotifers: Environmental and Endogenous Controls, Development, and Features for Predictable or Unpredictable Environments. Biol. Rev. Camb Philos. Soc 92 (2), 964–992. doi: 10.1111/brv.12264 27000555

[B22] Gladyshev E. A. Meselson M. Arkhipova I. R. (2008). Massive Horizontal Gene Transfer in Bdelloid Rotifers. Science 320 (5880), 1210–1213. doi: 10.1126/science.1156407 18511688

[B23] Hanington P. C. Forys M. A. Dragoo J. W. Zhang S. M. Adema C. M. Loker E. S. (2010). Role for a Somatically Diversified Lectin in Resistance of an Invertebrate to Parasite Infection. Proc. Natl. Acad. Sci. U. S. A. 107 (49), 21087–21092. doi: 10.1073/pnas.1011242107 21084634 PMC3000291

[B24] He P. Wang W. Sanogo B. Zeng X. Sun X. Lv Z.. (2017). Molluscicidal Activity and Mechanism of Toxicity of a Novel Salicylanilide Ester Derivative Against Biomphalaria Species. Parasit Vectors. 10 (1), 383. doi: 10.1186/s13071-017-2313-3 28793917 PMC5550999

[B25] Imai S. Miyazaki H. Nomura K. (1991). Trichodinid Species From the Gill of Cultured Japanese Eel, Anguilla Japonica, With the Description of a New Species Based on Light and Scanning Electron Microscopy. Eur. J. Protistol. 27 (1), 79–84. doi: 10.1016/S0932-4739(11)80430-X 23194613

[B26] Jose D. P. S. Paggi J. Collins P. Collins J. Graciela B. (2008). Water Quality and Zooplankton Composition in a Receiving Pond of the Stormwater Runoff From an Urban Catchment. J. Environ. Biol. 29 (5), 693–700. 19295067

[B27] Keiser J. Vargas M. Rubbiani R. Gasser G. Biot C. (2014). In Vitro and In Vivo Antischistosomal Activity of Ferroquine Derivatives. Parasit Vectors. 7:424. doi: 10.1186/1756-3305-7-424 25190030 PMC4164798

[B28] Kengne-Fokam A. C. Nana-Djeunga H. C. Djuikwo-Teukeng F. F. Njiokou F. (2016). Analysis of Mating System, Fecundity, Hatching and Survival Rates in Two Schistosoma Mansoni Intermediate Hosts (Biomphalaria Pfeifferi and Biomphalaria Camerunensis) in Cameroon. Parasit Vectors. 9:10. doi: 10.1186/s13071-015-1285-4 26739376 PMC4702333

[B29] Kumar S. Stecher G. Tamura K. (2016). MEGA7: Molecular Evolutionary Genetics Analysis Version 7.0 for Bigger Datasets. Mol. Biol. Evol. 33 (7), 1870–1874. doi: 10.1093/molbev/msw054 27004904 PMC8210823

[B30] Lardans V. Dissous C. (1998). Snail Control Strategies for Reduction of Schistosomiasis Transmission. Parasitol Today 14 (10), 413–417. doi: 10.1016/s0169-4758(98)01320-9 17040832

[B31] Lin D. Zeng X. Sanogo B. He P. Xiang S. Du S.. (2020). The Potential Risk of Schistosoma Mansoni Transmission by the Invasive Freshwater Snail Biomphalaria Straminea in South China. PloS Negl. Trop. Dis. 14 (6), e8310. doi: 10.1371/journal.pntd.0008310 PMC7302743 32511225

[B32] Looker D. L. Etges F. J. (1979). Effect of Schistosoma Mansoni Infection on Fecundity and Perivitelline Fluid Composition in Biomphalaria Glabrata. J. Parasitol. 65 (6), 880–885. doi: 10.2307/3280241 575549

[B33] Lu X. T. Gu Q. Y. Limpanont Y. Song L. G. Wu Z. D. Okanurak K.. (2018). Snail-Borne Parasitic Diseases: An Update on Global Epidemiological Distribution, Transmission Interruption and Control Methods. Infect. Dis. Poverty. 7 (1), 28. doi: 10.1186/s40249-018-0414-7 29628017 PMC5890347

[B34] Meier-Brook C. (1974). A Snail Intermediate Host of Schistosoma Mansoni Introduced Into Hong Kong. Bull. World Health Organ. 51 (6), 661. 4549615 PMC2366262

[B35] Meier M. Meier-Brook C. (1981). Schistosoma Mansoni: Effect on Growth, Fertility, and Development of Distal Male Organs in Biomphalaria Glabrata Exposed to Miracidia at Different Ages. Z Parasitenkd. 66 (2), 121–131. doi: 10.1007/BF00925719 7324545

[B36] Meyabeme E. A. Liess M. Duquesne S. (2010). Influence of Competing and Predatory Invertebrate Taxa on Larval Populations of Mosquitoes in Temporary Ponds of Wetland Areas in Germany. J. Vector Ecol. 35 (2), 419–427. doi: 10.1111/j.1948-7134.2010.00101.x 21175950

[B37] Mourao M. M. Dinguirard N. Franco G. R. Yoshino T. P. (2009). Role of the Endogenous Antioxidant System in the Protection of Schistosoma Mansoni Primary Sporocysts Against Exogenous Oxidative Stress. PloS Negl. Trop. Dis. 3 (11), e550. doi: 10.1371/journal.pntd.0000550 19924224 PMC2771906

[B38] Oliveira-Filho E. C. Paumgartten F. J. (2000). Toxicity of Euphorbia Milii Latex and Niclosamide to Snails and Nontarget Aquatic Species. Ecotoxicol. Environ. Saf. 46 (3), 342–350. doi: 10.1006/eesa.2000.1924 10903832

[B39] Picapedra P. Fernandes C. Baumgartner G. Sanches P. V. (2021). Zooplankton Communities and Their Relationship With Water Quality in Eight Reservoirs From the Midwestern and Southeastern Regions of Brazil. Braz. J. Biol. 81 (3), 701–713. doi: 10.1590/1519-6984.230064 32876161

[B40] Pila E. A. Gordy M. A. Phillips V. K. Kabore A. L. Rudko S. P. Hanington P. C. (2016). Endogenous Growth Factor Stimulation of Hemocyte Proliferation Induces Resistance to Schistosoma Mansoni Challenge in the Snail Host. Proc. Natl. Acad. Sci. U. S. A. 113 (19), 5305–5310. doi: 10.1073/pnas.1521239113 27114544 PMC4868488

[B41] Ranasinghe H. Amarasinghe L. D. (2020). Naturally Occurring Microbiota in Dengue Vector Mosquito Breeding Habitats and Their Use as Diet Organisms by Developing Larvae in the Kandy District, Sri Lanka. BioMed. Res. Int. 2020:5830604. doi: 10.1155/2020/5830604 33102582 PMC7578733

[B42] Reyes-Prieto M. Oceguera-Figueroa A. Snell S. Negredo A. Barba E. Fernandez L.. (2014). DNA Barcodes Reveal the Presence of the Introduced Freshwater Leech Helobdella Europaea in Spain. Mitochondrial DNA. 25 (5), 387–393. doi: 10.3109/19401736.2013.809426 23885897

[B43] Rozemberg B. Rey L. Pieri O. S. (1992). Fecundity of Biomphalaria Straminea and B. Glabrata in the Laboratory: A 12-Month Comparative Study. Mem Inst Oswaldo Cruz. 87 (Suppl 1), 223–232. doi: 10.1590/s0074-02761992000500042 1343791

[B44] Scherrer J. F. Chquiloff M. A. de Freitas J. R. (1976). [Comparative Study on the Reproduction in 4 Genetic Variations of Biomphalaria Glabrata (Say 1818) I. Fertility. Rev. Inst. Med. Trop. Sao Paulo. 18 (5), 315-321. 1006064

[B45] Soberon M. Lopez-Diaz J. A. Bravo A. (2013). Cyt Toxins Produced by Bacillus Thuringiensis: A Protein Fold Conserved in Several Pathogenic Microorganisms. Peptides. 41, 87–93. doi: 10.1016/j.peptides.2012.05.023 22691603

[B46] Stelzer C. P. (2009). Automated System for Sampling, Counting, and Biological Analysis of Rotifer Populations. Limnol Oceanogr Methods 7, 856–864. doi: 10.4319/lom.2009.7.856 21151824 PMC2999893

[B47] Szydlowski L. Boschetti C. Crisp A. Barbosa E. G. Tunnacliffe A. (2015). Multiple Horizontally Acquired Genes From Fungal and Prokaryotic Donors Encode Cellulolytic Enzymes in the Bdelloid Rotifer Adineta Ricciae. Gene 566 (2), 125–137. doi: 10.1016/j.gene.2015.04.007 25863176

[B48] Wang W. Mao Q. Yao J. Yang W. Zhang Q. Lu W.. (2018). Discovery of the Pyridylphenylureas as Novel Molluscicides Against the Invasive Snail Biomphalaria Straminea, Intermediate Host of Schistosoma Mansoni. Parasit Vectors. 11 (1), 291. doi: 10.1186/s13071-018-2868-7 29743096 PMC5944108

[B49] Wang L. Wu X. Li X. Zheng X. Wang F. Qi Z.. (2020). Imported Schistosomiasis: A New Public Health Challenge for China. Front. Med. (Lausanne). 7, 553487. doi: 10.3389/fmed.2020.553487 33195303 PMC7642816

[B50] Wei G. Lai Y. Wang G. Chen H. Li F. Wang S. (2017). Insect Pathogenic Fungus Interacts With the Gut Microbiota to Accelerate Mosquito Mortality. Proc. Natl. Acad. Sci. U. S. A. 114 (23), 5994–5999. doi: 10.1073/pnas.1703546114 28533370 PMC5468619

[B51] Xu X. Chen T. Xie A. Yang X. Wei X. (2021). Chronic Effects of Bromate on Sexual Reproduction of Freshwater Rotifer Brachionus Calyciflorus. Bull. Environ. Contam. Toxicol. 106 (2), 270–277. doi: 10.1007/s00128-021-03103-z 33471188

[B52] Xu K. Meng F. Song W. (2000). Scanning Electron Microscopic Observations on the Histopathology of Trichodiniasis of the Mariculture Fish, Lateolabrax Japonicus (In Chinese). J. Ocean Univ. Qingdao (03), 418–422. doi: 10.16441/j.cnki.hdxb.2000.03.009

[B53] Xu K. Song W. Warren A. (1999). Trichodinid Ectoparasites (Ciliophora: Peritrichida) From the Gills of Cultured Marine Fishes in China, With the Description of Trichodinella Lomi N. Sp. Syst. Parasitol. 42 (3), 219–227. doi: 10.1023/a:1006067005936 10613540

[B54] Xu L. Xu M. Sun X. Xu J. Zeng X. Shan D.. (2019). The Genetic Basis of Adaptive Evolution in Parasitic Environment From the Angiostrongylus Cantonensis Genome. PloS Negl. Trop. Dis. 13 (11), e7846. doi: 10.1371/journal.pntd.0007846 PMC6871775 31751335

[B55] Yan D. C. Dong S. L. Huang J. Yu X. M. Feng M. Y. Liu X. Y. (2004). White Spot Syndrome Virus (WSSV) Detected by PCR in Rotifers and Rotifer Resting Eggs From Shrimp Pond Sediments. Dis. Aquat. Organ. 59 (1), 69–73. doi: 10.3354/dao059069 15212294

[B56] Yang Y. Cheng W. Wu X. Huang S. Deng Z. Zeng X.. (2018). Prediction of the Potential Global Distribution for Biomphalaria Straminea, an Intermediate Host for Schistosoma Mansoni. PloS Negl. Trop. Dis. 12 (5), e6548. doi: 10.1371/journal.pntd.0006548 PMC5993297 29813073

[B57] Yang G. J. Li W. Sun L. P. Wu F. Yang K. Huang Y. X.. (2010). Molluscicidal Efficacies of Different Formulations of Niclosamide: Result of Meta-Analysis of Chinese Literature. Parasit Vectors. 3, 84. doi: 10.1186/1756-3305-3-84 20819229 PMC2944309

[B58] Yan D. Yan J. Deng Y. (2007). Advances in the Research of Rotifer Diseases (In Chinese). Reservoir Fisheries (05), 105–107. doi: 10.3969/j.issn.1003-1278.2007.05.044

[B59] Zhu G. L. Tang Y. Y. Limpanont Y. Wu Z. D. Li J. Lv Z. Y. (2019). Zoonotic Parasites Carried by Invasive Alien Species in China. Infect. Dis. Poverty. 8 (1), 2. doi: 10.1186/s40249-018-0512-6 30621776 PMC6325848

